# Editorial: Phenotypes of myasthenia gravis

**DOI:** 10.3389/fneur.2022.1025183

**Published:** 2022-09-14

**Authors:** Hai-Feng Li, Nils Erik Gilhus, Huan Yang, Xiangjun Chen

**Affiliations:** ^1^Department of Neurology, Xuanwu Hospital, Capital Medical University, Beijing, China; ^2^Department of Neurology, Haukeland University Hospital, Bergen, Norway; ^3^Department of Clinical Medicine, University of Bergen, Bergen, Norway; ^4^Department of Neurology, Xiangya Hospital, Central South University, Changsha, China; ^5^Department of Neurology, Huashan Hospital, Fudan University, Shanghai, China; ^6^Human Phenome Institute, Fudan University, Shanghai, China

**Keywords:** myasthenia gravis, phenotype, personalized treatment, mechanism, immunotherapy

Disease phenotypes are observable and recognizable traits of diseases, which are not limited to hereditary diseases. A single essential feature or a specific combination of features in a disease can be defined using qualitative and quantitative descriptions, with the goal of understanding the full spectrum of disease phenotypes. This will lay the foundation for a reasonable diagnostic process, for assessing illness severity and treatment efficacy, and for identifying the individualized characteristics of patients to guide a precise personalized treatment.

Myasthenia gravis (MG) is a prototypical autoimmune disease with well-defined autoantibodies that target the neuromuscular junction. However, MG exhibits a high degree of phenotypic heterogeneity. Demographic characteristics, extent of muscle involvement, disease progression, presence and level of pathogenic antibodies, immunologic profiles, quantitative measurements of severity, comorbidities, subgroup classification, drug efficacy and long-term stability are all phenotypic characteristics that differ among individual patients. This special topic, including 13 original research articles, two brief research reports, two reviews, and one opinion article, all relevant to the above-mentioned phenotypic characteristics, contributes to an improved understanding and assessment of MG phenotypes.

## Phenotypic description

A comprehensive description of phenotypic characteristics provides an integrative understanding of the disease and highlights the clinical features that should be paid attention in clinical practice.

Short-term and long-term prognosis after a first acute dyspnea episode that occurred 12 (4~34.5) months after disease onset were reported in a study of 86 MG patients. Early-onset MG and precipitating respiratory infection were found as independent risk factors for progression to myasthenic crisis, which occurred in 41.9% of the included patients. However, with proper immunosuppressive therapy, the patients had an overall good prognosis (Huang et al.). In a study of 796 MG patients naïve to immune therapies, ≥1 concurrent autoimmune diseases were found in 11.6%. Compared to the general population, a significantly higher incidence of various autoimmune diseases was found, especially for hyperthyroidism, immune thrombocytopenic purpura, autoimmune hemolytic anemia, autoimmune hepatitis, and polymyositis. MG patients with concurrent autoimmune diseases were predominantly female, younger at MG onset, and they seldom had MuSK antibodies. Furthermore, they tended to have a mild clinical presentation of MG, including a lower proportion of previous myasthenic crisis and a higher proportion of MGFA Class I at onset (Shi, Huan et al.). Thymoma has a high frequency of concurrent autoimmune diseases, and MG in particular. Previous studies indicate that there is a difference in the concurrent autoimmune disease profile between MG patients with and without thymoma (1, 2). Shi, Huan et al. found that thymoma was less common in MG patients with concurrent autoimmune diseases.

With the increased use of immune-checkpoint inhibitors (ICIs) for cancer treatment, the incidence of neurological immune-related adverse events is growing. ICI-related MG (irMG) is relatively common and has a high fatality rate (3). In a case series combined with a systematic review, 63 irMG patients and 380 idiopathic MG patients were compared. Higher MGFA class and higher QMGS (i.e., more severe disease) were observed in irMG patients compared to idiopathic MG. More irMG patients had concurrent myositis or myocarditis. An unfavorable disease outcome was found in 35% of the irMG patients. Myocarditis, higher MGFA class and QMG score were associated with an unfavorable disease outcome in irMG patients (Shi, Tan et al.).

The International Consensus Guidance for Management of MG calls for the latest evidence relevant to the management of MG to be assessed (4). Some phenotypic subgroups have been little studied. Studies focusing on the natural history and treatment response in patients of very early and very late onset ages, and with ocular onset, are collected in this special topic (Bi et al.; Zhao et al.; Zheng et al.; Zhou et al.). The studies attempt to define indicators of treatment response and prognosis applying real world data and using a retrospective design. Furthermore, phenotypic differences in juveniles with ocular manifestations of MG in different populations were discussed in detail and with a special focus on pathogenic mechanisms and treatment responses in a comprehensive review (Heckmann et al.).

Healthcare resource utilization (HCRU) and costs associated with generalized MG were reported in a study of 41,940 patients of the United States. Mean HCRU and costs were higher for newly diagnosed patients and patients with exacerbation events. For patients who experienced MG crisis, HCRU and costs markedly increased during the 12 months immediately before the crisis event compared with the two preceding years. The costs increased further during the 12 months following the index crisis event (Phillips et al.). This study provided valuable data on health economics in MG patients of generalized phenotype.

## Phenotypic biomarkers

Autoantibodies in patients with MG can target all subunits of the AChR at both their extracellular and intracellular regions. In one study, a combination of immunoadsorption with cell-based assays (CBA) was used to examine the specificity of the autoantibodies against the extracellular parts of AChR molecule in AChR antibody positive patients defined by RIPA. Antibodies against intracellular region were found probably not related to neuromuscular transmission impairment, although a detailed analysis was not available. Moreover, the autoantibodies were divided into distinct groups based on their target, highly relevant for disease severity. The antibodies against non-α1 epitopes were found in patients with a milder disease, and they were inversely correlated with MGFA class. A combination of RIPA and CBA is recommended by the authors for the follow-up of MG. The former method is to be used for the quantification of the antibodies and the latter for the identification of fluctuations in culprit antibodies (Michail et al.). This study represents an important advance in the understanding of AChR antibodies in MG. However, the generation mechanism and diagnostic value of anti-intracellular region antibodies remain to be elucidated.

Pathogenic and MG-associated antibodies represent main phenotypic variables in MG subgroup classification with the purpose of individualized or stratified treatment. Whether antibodies combined with clinical variables are useful in deciding optimal therapy was examined in a study of 188 treatment-naïve generalized MG patients who were single AChR antibody positives, dual AChR and LRP4 antibody positives, and dual AChR and titin antibody positives. Patients with AChR plus titin antibodies had more severe MG and progressed faster than those with AChR plus LRP4 antibodies and those with only AChR antibodies. However, all patients responded well to immunotherapy and had relatively good prognosis regardless of the three antibody groups (Chen et al.). MG patients with MuSK antibodies represent a distinct subgroup. Originally regarded as a severe MG, there are now reports of patients with a relatively benign course, or with overlapping phenotypes between MuSK-MG and AChR-MG (5). In a study of 69 MuSK-MG patients, comparison of clinical features and outcomes at 3, 6, and 12 months after onset were conducted among those with different onset age (early-onset, late-onset, and very-late-onset). The very-late-onset subgroup had the highest frequency of limb, bulbar and respiratory involvement, which might prompt earlier usage of potent immunosuppressive therapy. Most MuSK-MG patients benefited from rituximab treatment regardless of age at onset (Zhou et al.).

Immunologic biomarkers such as LINC00680, a long non-coding RNA, were found associated with the QMG score in a small cohort of MG patients (Liu et al.). More researches on the association between immunological profiles and treatment effects and prognosis of MG are needed.

## Phenotypic correlation on treatment response

Glucocorticoid (GC) represents the mainstay of MG treatment. However, prolonged usage of high-dose GC leads to various adverse effects. Therefore, there is consensus that low-dose GC is the aim for long-term maintenance of long-term therapy. Clinical factors related to relapses during GC tapering or after withdrawal were investigated in a study of 125 MG patients who were stable on GC monotherapy. Relapse during the steroid reduction was found to be associated with drug-reducing speed. Furthermore, relapses were more prevalent in patients with onset symptoms of bulbar weakness (Su et al.). In a study of 149 GC-resistant childhood-onset MG patients, 75.8% responded well to tacrolimus. One month after initiating tacrolimus, QMG and ADL scores had improved and the prednisone dose was reduced. QMG and ADL scores continued to improve throughout the study. The prednisone treatment was eventually stopped in 78.8% of the patients. Thymus pathology and pre-intervention status were found to be independent predictors of tacrolimus efficacy (Bi et al.).

Predictors of secondary generalization in patients with vary late onset MG were explored in 69 patients. Absence of immunotherapy was found as the only predictor of secondary generalization in those with pure ocular onset (Zhao et al.). In a study of 53 MG patients with MuSK antibodies, the relapse rate was significantly lower in patients receiving GC combined with other immunosuppressants than in those with only GC. Of all potential associated factors, only the use of additional immunosuppressants was associated with a lower relapse risk (Tan et al.). Among 70 very late onset MG patients, no significant differences in outcomes were observed between those receiving tacrolimus treatment alone and those with tacrolimus combined with GC. Nor did the outcome differ between the tacrolimus group and the group that had never used tacrolimus or used tacrolimus for <3 months. No significant associations were found between tacrolimus administration and clinical outcomes. Although high quality of life was observed in patients treated with tacrolimus, which is better over another in using directly tacrolimus mono-therapy or combining GCs first to stabilize the disease and then taking tacrolimus alone for maintenance therapy is not clear (Zheng et al.).

Treatment resistance to GC is an important phenotypic variable in the treatment of MG. Presently, treatment-resistant patients can only be defined retrospectively. To what degree early treatment response predicts long-term refractoriness is unknown. In an integrative review, definition of GC resistance in MG was discussed in relevance to potential mechanisms, including the underlying MG pathology explaining no response to GC, the susceptibility to GC adverse effects that compromise the ability to achieve therapeutic doses, and the phenotypic and genetic variations that limit the response to GC. Moreover, the authors emphasized that neither patient nor clinician should be content with just an improvement from a poor baseline and with still considerable disability. The aim should be expecting a situation close to minimal manifestation status (MMS) (Kaminski and Denk). Some generalized MG patients are difficult to treat, but true non-responsive and refractory disease hardly occurs. However, extraocular muscles are vulnerable to be impaired in shorter periods due to functional denervation. Hence, definitions for difficult-to-treat or refractory generalized MG do not apply to ocular involvement in MG. Based on the treatment outcomes of extraocular muscles in MG and presumed pathogenic mechanisms, a definition for treatment-resistant ophthalmoplegia was proposed in a comprehensive review (Heckmann et al.).

## Methods to assess MG phenotypes

Measurements of disease status and criteria of treatment response represent important phenotypic variables for MG. In one study, the items in MG-QOL focusing on work skills were found to be less relevant for very late onset MG patients since the majority were retired (Zheng et al.). Once MG is well-controlled with immunotherapy, many patients stop pyridostigmine, may take it only when fatigued, or take 1~2 tablets daily out of habit and for a sense of security (6). The influence of taking pyridostigmine on determination of the post-intervention status (PIS) categories was reported. In the same study, with a standardized flowchart and working definitions for the real-time and sustained (for 3, 6, and 12 months) PIS categories, sustainability of the R/MM status was confirmed in a prospective cohort of 376 patients with mild to moderate disease. The QMG, MG-ADL and MG-QOL15 scores among patients belonging to each real-time and sustained PIS category at baseline and follow-ups were significantly different, ranking as R < MM < SI. The GC and pyridostigmine doses decreased with time and ranked as R < MM < SI. This indicates that R/MM represents an immunologic stable state (Jiang et al.). Treatment response can be expressed as percentage of change from baseline (relative criterion) in autoimmune diseases (7). In a retrospective cohort of 257 immunotherapy-native MG patients, response to a 3-month standardized GC treatment was evaluated with commonly-used absolute criteria. Cut-offs for relative criteria were generated using a receiver-operating characteristic curves both for the whole cohort and in patients stratified for pre-treatment QMG score. The consistency between the absolute criterion and the finally-selected relative criterion was substantial in the whole cohort, but was moderate in severe group. Some severe patients were classified as responsive with absolute criterion while as unresponsive with relative criterion. This finding is consistent with clinical experience (Li et al.).

Although evaluation of MG status by the clinicians is important in daily practice, patient-reported information and patient experience provide important knowledge on the disease itself and its management. MG research needs the input from patients who have experienced various symptoms, examinations and therapies, as well as multiple consequences of having MG. MG patients know from experience the needs for a precise diagnosis and better treatment, for correct information and more knowledge. The linguistic shift from “patient” to “user” reflects a change in ideology of medical research. The active participation of MG patients may bring something new into a research project, this also being true for subgroups such as children, pregnant women, the very old, and immigrants. In a thought-provoking opinion article, patient involvement was discussed in relevance to the phenotypic variation of MG (Gilhus et al.).

## Author contributions

All authors listed have made a substantial, direct, and intellectual contribution to this editorial and approved it for publication.

## Conflict of interest

The authors declare that the research was conducted in the absence of any commercial or financial relationships that could be construed as a potential conflict of interest.

## Publisher's note

All claims expressed in this article are solely those of the authors and do not necessarily represent those of their affiliated organizations, or those of the publisher, the editors and the reviewers. Any product that may be evaluated in this article, or claim that may be made by its manufacturer, is not guaranteed or endorsed by the publisher.
